# Polybromides Attraction‐Repulsion Regulation Promotes High‐Performance Zinc–Bromine Static Battery

**DOI:** 10.1002/advs.77018

**Published:** 2026-09-06

**Authors:** Jiamin Tang, Ling He, Lei Liu, Huanwei Li, Haichuan He, Jie Wang, Zhengxin Zhu

**Affiliations:** ^1^ School of Chemistry and Chemical Engineering University of South China Hengyang China

**Keywords:** attraction‐repulsion regulation, fast kinetics, high reversibility, shuttle effect, zinc‑bromine battery

## Abstract

Aqueous zinc‑bromine (Zn–Br) static batteries that employ bromine complexing agents offer excellent reversibility and robust cyclability, holding great promise for grid‑scale energy storage. However, the sluggish kinetics of the resulting solid complexes hinder their practical applications. Herein, we develop an attraction‐repulsion regulation relying on choline cations (Ch^+^) and sulfate anions (SO_4_^2−^) to confine the polybromides (Br_3_^−^) and enable fast kinetics. Experiments and theoretical calculations confirm that the strong bonding effect of Ch^+^ and high negative charge density of SO_4_^2−^ prevent the Br_3_^−^ from dissolving and shuttling into the electrolyte. Consequently, the Zn–Br static battery with the optimal electrolyte achieves a reversible capacity of 166 mAh g^−1^ (based on the mass of activated carbon) with an average discharge voltage of 1.73 V at 0.2 A g^−1^, while maintaining a remarkable rate capability with a high discharge capacity of 99 mAh g^−1^ at 8 A g^−1^ and a long lifespan over 15 000 cycles with a capacity retention of 84.9% at 5 A g^−1^. Moreover, pouch cell under a high areal capacity of 8.3 mAh cm^−2^ exhibits a discharge capacity of 365 mAh after 100 cycles at 0.05 A g^−1^. This work establishes a critical pathway toward the subsequent development of Zn–Br static battery.

## Graphical abstract

Br_3_^−^ attraction‐repulsion regulation enhances the reversibility and reaction kinetics of Zn–Br static batteries. Coin cell shows a high average discharge voltage of 1.73 V and a fast charge/discharge rate up to 32 mA cm^−2^. Pouch cell with a high areal capacity of 8.3 mAh cm^−2^ achieves a real capacity of 365 mAh after 100 cycles.

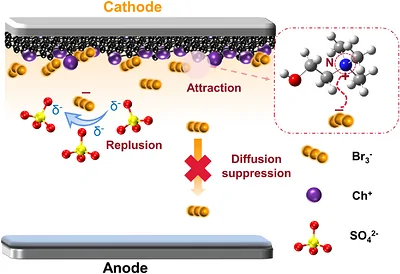

## 1 Introduction

Driven by the escalating global demand for efficient and scalable energy storage, advanced battery technologies have emerged as a critical research priority [1]. An ideal battery energy storage system is one that concurrently offers high safety, low cost, long cycle lifetime, high energy density, and high power density to meet the diverse application scenarios [2, 3, 4]. Despite their longstanding dominance in the energy storage landscape, lithium‐ion batteries are constrained by inherent limitations, including the scarcity of lithium resources and the usage of flammable electrolytes [5, 6]. Leveraging their intrinsic safety, rechargeable aqueous batteries present a compelling advantage over non‐aqueous alternatives [7, 8, 9]. In particular, aqueous zinc batteries (AZBs) are considered a highly promising technology for grid‐scale energy storage by virtue of the high theoretical capacity (820 mAh g^−1^) and low redox potential (−0.762 V vs. SHE) of Zn anode [10, 11, 12]. The selection of suitable cathode materials is paramount to determining the overall battery performance [8]. So far, researchers have explored various cathode materials for AZBs, including transition metal‐based compounds [13], Prussian blue analogues [14], and organic materials [15]. However, traditional cathodes mainly rely on the Zn^2+^/H^+^ co‐intercalation mechanism for energy storage, which easily causes structural collapse of materials, thereby lowering the battery stability [16].

Currently, halogen cathodes (e.g., iodine and bromine) have attracted extensive interest in energy storage applications, owing to their high‐efficiency conversion reaction that effectively avoids the structural collapse commonly observed in intercalation cathodes [17, 18, 19, 20]. Among them, zinc‐bromine (Zn–Br) flow batteries present a highly attractive option due to their high theoretical specific capacity (335 mAh g^−1^), high redox potential (1.08 V vs. SHE), and cost‐effectiveness of bromine cathode [21, 22, 23]. During battery charging, Br_2_ generated at the cathode can further react with Br^−^ in aqueous electrolyte to produce highly soluble polybromides (Br_3_^−^) [22]. Although the formation of highly soluble Br_3_^−^ is beneficial for battery kinetics, their shuttle effect leads to severe side reactions in the Zn anode, thereby causing self‐discharge and low Coulombic efficiency (CE) [24]. Specifically, Zn–Br flow batteries require a circulation pump system and ion‐selective membrane to prevent the crossover of Br_3_^−^, thereby leading to the lower energy density and higher cost [25, 26, 27]. In contrast, Zn–Br static batteries eliminate the need for extra equipment, which significantly increases the battery energy density [28, 29, 30, 31]. However, Zn–Br static batteries still involve the formation of soluble Br_3_^−^, compromising their cycling stability.

To address the above challenge, tremendous progress has been made in the optimization of electrode materials and electrolytes for Zn–Br static batteries [21, 29, 31, 32, 33, 34, 35]. In the field of cathode materials, researchers have focused on designing novel catalysts for confining the migration of Br_3_^−^ from electrode to electrolyte [34, 36, 37, 38, 39]. For instance, Lin and coworkers designed a highly conductive MXene for enhancing the adsorption and conversion of Br_3_^−^ [38]. However, the host material provides limited catalytic sites in Br_3_^−^, leading to the low discharge capacity. Through electrolyte engineering, deep eutectic electrolytes and ionic liquid modifications have been developed as a promising strategy [31, 40, 41]. Notably, commonly used bromine complexing agents with a high atomic charge of N^+^ serve to convert Br_3_^−^ into insoluble complexes, which effectively suppress the dissolution and diffusion of Br_3_^−^ [37, 40, 42, 43]. For instance, Yoo and coworkers simultaneously introduced the deep eutectic solvent and tetrabutylammonium bromide (TBABr) additive into the electrolyte of Zn–Br battery to confine the dissolution of Br_3_^−^ [40]. However, the produced solid complexes during battery charging often lead to sluggish kinetics and low discharge voltage. Therefore, a key design goal for the electrolyte is to suppress the crossover of Br_3_^−^ and enable the rapid bromine redox kinetics.

In our work, we introduce the choline cations (Ch^+^) into zinc sulfate (ZnSO_4_) electrolyte, which facilitates both rapid and reversible Br_3_^−^ conversion reaction in Zn–Br static batteries through the attraction‐repulsion regulation. After optimizing the ZnSO_4_ concentration, the battery reversibility can be further improved by the disruption of hydrogen bond (H─bond) network among water molecules. Experimental analysis demonstrates that Ch^+^ restrict Br_3_^−^ to the cathode side by their strong electrostatic attraction and SO_4_^2−^ suppress their diffusion into the electrolyte through strong electrostatic repulsion. In situ ultraviolet‐visible (UV–vis) spectroscopy confirms that the migration of Br_3_^−^ is well suppressed during battery charging. As a result, the Zn–Br static battery with the optimal electrolyte shows a reversible capacity of 166 mAh g^−1^ (based on the mass of activated carbon) and an average discharge voltage of 1.73 V at 0.2 A g^−1^. Meanwhile, the Zn–Br static battery exhibits excellent rate capability (99 mAh g^−1^ at 8 A g^−1^) and cycling stability (84.9% capacity retention after 15 000 cycles at 5 A g^−1^). More importantly, a practical pouch cell based on a high areal capacity of 8.3 mAh cm^−2^ delivers a discharge capacity of 365 mAh after 100 cycles at 0.05 A g^−1^.

## 2 Results and Discussion

**FIGURE 1:**
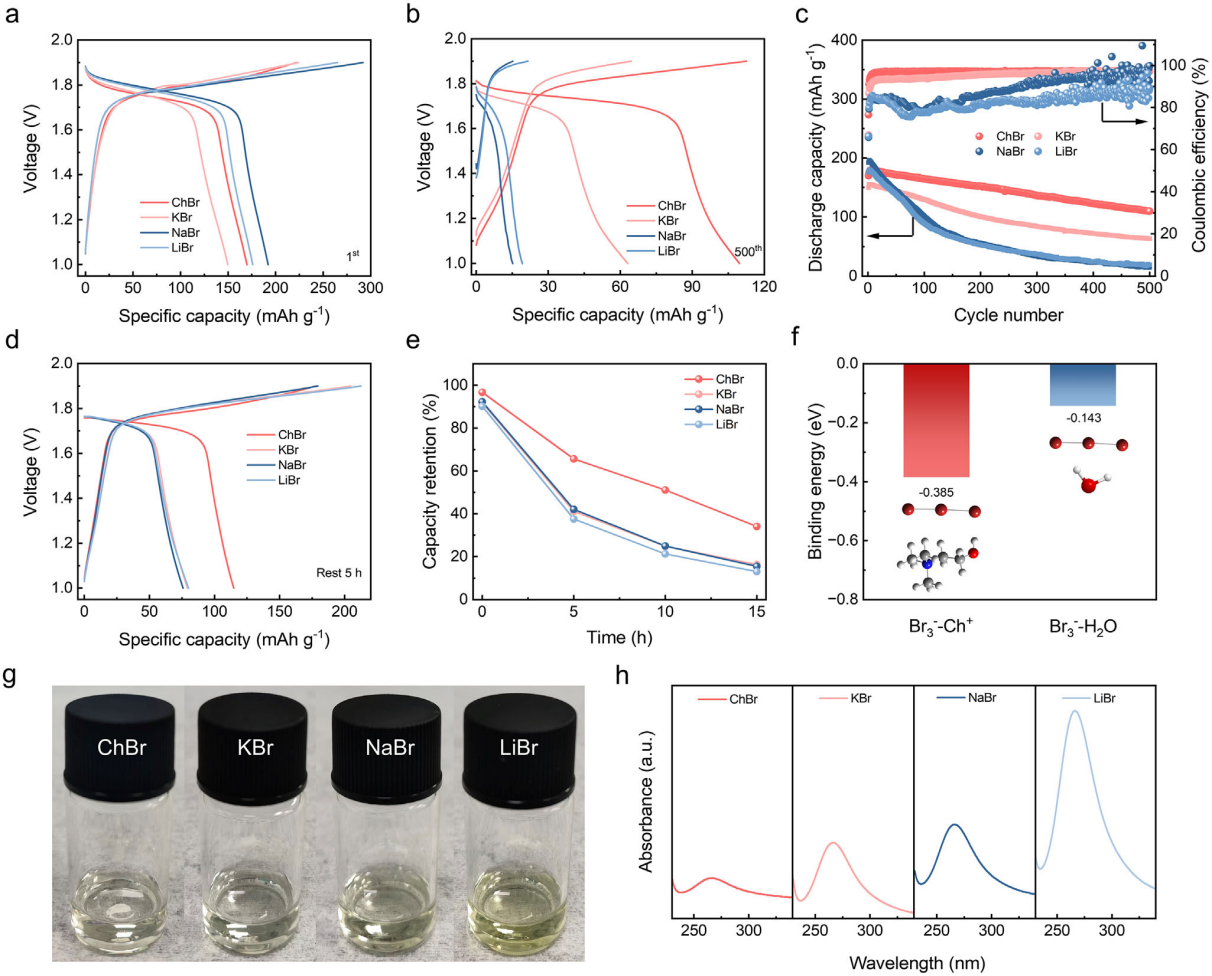
(a) GCD curves of first cycle, (b) GCD curves of 500th cycle, and (c) cycling performance of Zn–Br static battery with various Br‐based electrolytes at a current density of 0.5 A g^−1^. (d) GCD curves after self‐discharge 5 h and (e) capacity retention after self‐discharge 15 h of Zn–Br static battery with various Br‐based electrolytes at a current density of 0.5 A g^−1^. (f) DFT calculations of binding energy of Br_3_^−^‐Ch^+^ and Br_3_^−^‐H_2_O. (g) Digital photo and (h) UV–vis spectra of the tested Br‐based electrolytes after 10 cycles.

In Zn–Br static batteries, the Br_3_^−^ generated during charging often causes a detrimental shuttle effect [41, 44]. The selection of an appropriate electrolyte to inhibit the crossover of Br_3_^−^ is therefore critical to advancing high‐performance Zn–Br static batteries. To evaluate the influence of cations, a series of electrolytes was formulated by combining the 1 m ZnSO_4_ with 0.5 m of various Br salts (e.g., ChBr, KBr, NaBr, and LiBr). The activated carbon was employed as the cathode in Zn–Br static batteries for the following testing. We assembled Zn–Br static batteries using various Br‐based electrolytes and collected galvanostatic charge‐discharge (GCD) curves. As shown in Figure 1a, the battery with ChBr electrolyte demonstrates an initial discharge capacity of 170 mAh g^−1^ at 0.5 A g^−1^ (based on the mass of activated carbon unless otherwise stated), which shows a minor deviation compared to other Br‐based electrolytes. After 500 cycles, the battery with ChBr electrolyte still exhibits a high discharge capacity of 110 mAh g^−1^ (Figure 1b). In contrast, the batteries with other Br‐based electrolytes all deliver fast capacity decay above 58% after 500 cycles. In addition, the Zn–Br static battery with ChBr electrolyte shows good reversibility, achieving a higher average CE of 97.1% than that of other Br‐based electrolytes after 500 cycles (Figure 1c). In addition to the deterioration of cycling stability, the dissolution and diffusion of Br_3_^−^ into aqueous electrolyte also result in a severe self‐discharge phenomenon in the Zn–Br static batteries [30]. Accordingly, we further evaluated the self‐discharge performance of Zn–Br static battery with various Br‐based electrolytes. The assembled Zn–Br static batteries were first cycled at 0.5 A g^−1^ for 10 cycles, followed by the self‐discharge tests in varying durations. As shown in Figure 1d, the battery with ChBr electrolyte exhibits a high‐capacity retention of 65.6% after self‐discharge 5 h, which is significantly superior to that of other Br‐based electrolytes (e.g., 41.2% capacity retention with KBr electrolyte). After self‐discharge 10 and 15 h, the Zn–Br static battery with ChBr electrolyte still delivers the highest capacity retention compared to other Br‐based electrolyte systems (Figure 1e). These results demonstrate the stable conversion of the Br^−^/Br_3_^−^ chemistry in the ChBr electrolyte.

To illustrate the suppressive effect of Ch^+^ on Br_3_^−^, we conducted the theoretical calculations using density functional theory (DFT). As shown in Figure 1f, the binding energy between the Ch^+^ and Br_3_^−^ is −0.385 eV, which is significantly more negative than that between the H_2_O and Br_3_^−^ (−0.143 eV). Notably, a more negative binding energy represents a stronger intermolecular interaction. The result indicates that the produced Br_3_^−^ can be effectively adsorbed by Ch^+^, thus inhibiting their shuttle effect. To visualize the Br_3_^−^ dissolution, we collected the tested electrolytes in Zn–Br static batteries after 10 cycles. No obvious color change is observed in the ChBr electrolyte, while the other Br‐based electrolytes all show a slight yellow color (Figure 1g). To further understand the shuttle behavior in various Br‐based electrolytes, ex situ UV–vis spectroscopy was conducted to examine the presence of Br_3_^−^. Figure 1h presents the absorption peak of Br_3_^−^, which is located at ∼266 nm. In the ChBr electrolyte, the absorption peak intensity of Br_3_^−^ is significantly lower than that of other Br‐based electrolytes, indicating effective Br_3_^−^ inhibition during battery cycling. Thus, ChBr as the optimal Br salt is selected for subsequent experiments.

**FIGURE 2:**
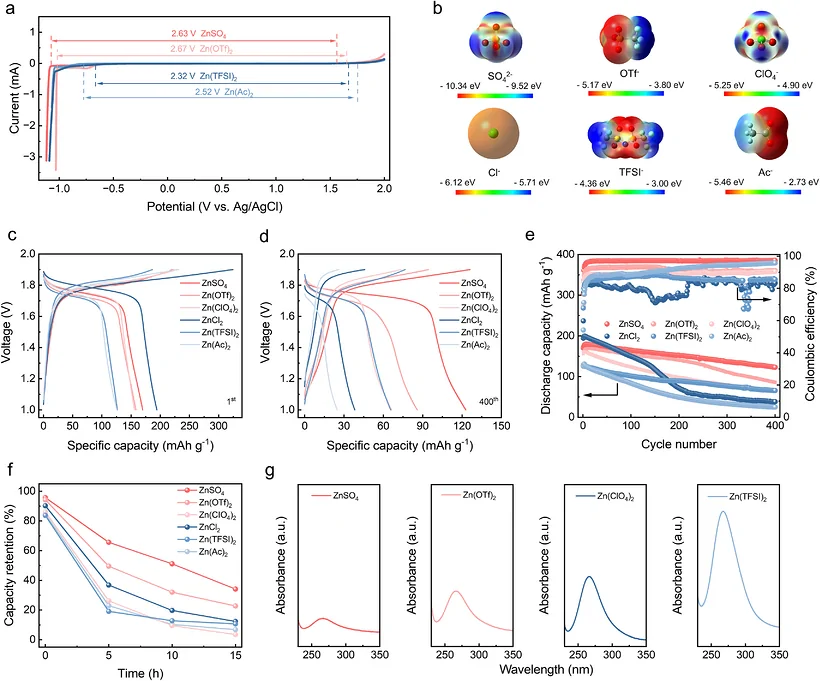
(a) LSV curves of various Zn‐based electrolytes at a scan rate 5 mV s^−1^. (b) ESP of various anions. (c) GCD curves of first cycle, (d) GCD curves of 400th cycle, and (e) cycling performance of Zn–Br static battery with various Zn‐based electrolytes at a current density of 0.5 A g^−1^. (f) Capacity retention after self‐discharge 15 h of Zn–Br static battery with various Zn‐based electrolytes at a current density of 0.5 A g^−1^. (g) UV–vis spectra of the tested Zn‐based electrolytes after 10 cycles.

To evaluate the effect of different anions on Br cathode, a series of electrolytes was prepared by combining the 0.5 m ChBr with 1 m Zn salts (e.g., ZnSO_4_, Zn(OTf)_2_, Zn(ClO_4_)_2_, ZnCl_2_, Zn(TSFI)_2_, and Zn(Ac)_2_). Figure 2a presents the linear sweeping voltammetry (LSV) curves of various Zn‐based electrolytes. It is obvious that both ZnSO_4_ and Zn(OTf)_2_ deliver a wide working voltage window of 2.63 and 2.67 V, respectively. In contrast, both Zn(Ac)_2_ and Zn(TFSI)_2_ exhibit a narrow working voltage window (2.52 and 2.32 V, respectively), which easily deteriorates the battery performance caused by H_2_ and O_2_ evolution. To understand the effect of anion, we calculated the electrostatic potential (ESP) of different anions. As shown in Figure 2b, the ESP value of SO_4_^2−^ keeps a range from −10.34 to −9.52 eV, which is significantly more negative than that of other anions (e.g., OTf^−^, ClO_4_^−^, Cl^−^, TFSI^−^, and Ac^−^). The result reveals that SO_4_^2−^ imposes strong electrostatic repulsion on Br_3_^−^ and displays a high affinity for H_2_O, thereby suppressing the solubility of Br_3_^−^ in the aqueous electrolyte. To verify the optimal ZnSO_4_ electrolyte for Zn–Br static batteries, various Zn‐based electrolytes were assembled into a coin cell for electrochemical testing. Compared to that in ZnSO_4_ electrolyte, Zn–Br static batteries experience a more rapid capacity decay in other Zn‐based electrolytes after 400 cycles (Figure 2c–e). Meanwhile, Zn–Br static battery with the ZnSO_4_ electrolyte achieves an average CE of 97.1% over 400 cycles, which is considerably higher than that of other Zn‐based electrolytes, thereby confirming its superior reversibility (Figure 2e). In ZnSO_4_ electrolyte, the self‐discharge of Zn–Br static battery is much slower than that in other Zn‐based electrolytes (Figure 2f). After resting at open circuit for 5 h, the discharge capacity decays from 178 to 115 mAh g^−1^ in ZnSO_4_ electrolyte, whereas after self‐discharge in other Zn‐based electrolytes, the remaining capacity is less than 74 mAh g^−1^ (Figure S1). To reveal the impact of Zn salts in inhibiting the dissolution and diffusion of Br_3_^−^, ex situ UV–vis spectroscopy was conducted to investigate the tested Zn‐based electrolytes after 10 cycles. Obviously, the ZnSO_4_ electrolyte shows negligible color change, but other Zn‐based electrolytes exhibit a slight yellow color (Figure S2). UV–vis spectroscopy spectra show that the absorption peak intensity of Br_3_^−^ in ZnSO_4_ electrolyte is significantly lower than that in other Zn‐based electrolytes. Therefore, we identify ZnSO_4_ and ChBr as the optimal electrolyte system, enabling Zn–Br static battery with high reversibility.

**FIGURE 3:**
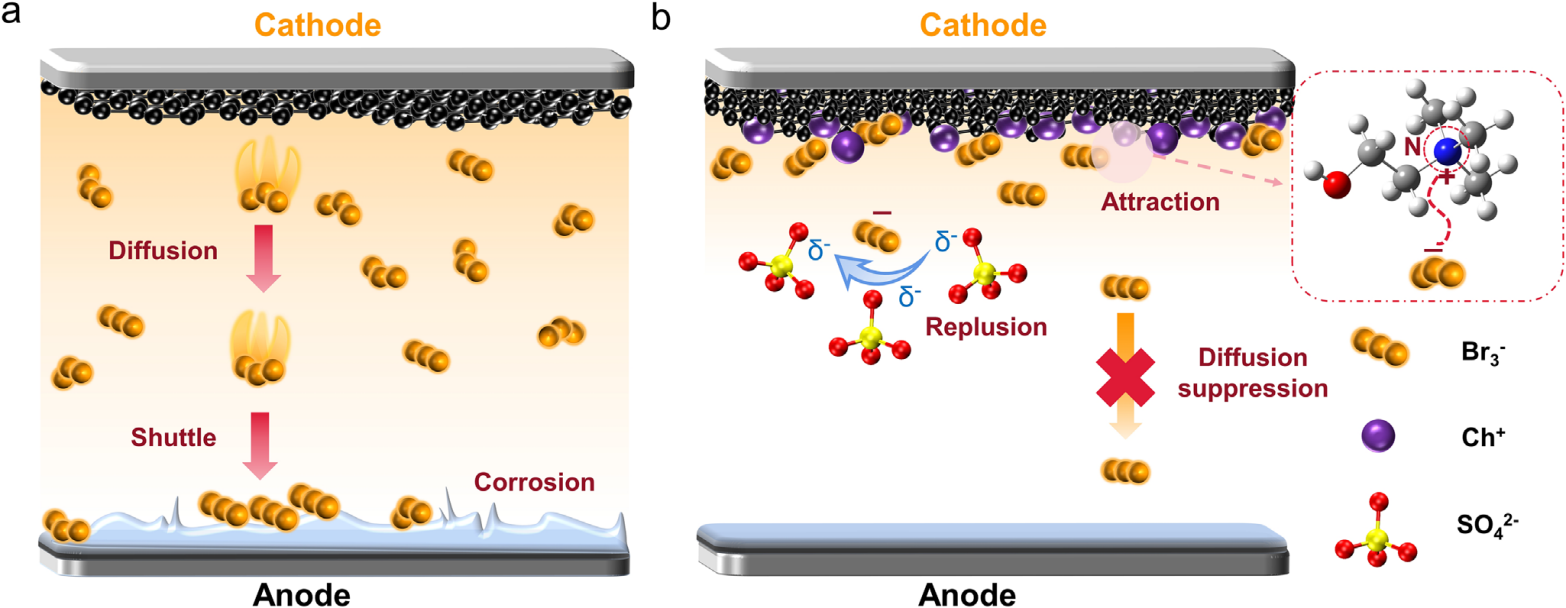
(a) Schematic diagram of shuttle effect of Br_3_^−^ without Ch^+^ and SO_4_^2−^ electrolyte. (b) Schematic diagram of diffusion suppression of Br_3_^−^ with Ch^+^ and SO_4_^2−^ electrolyte.

The electrochemical behavior of Zn–Br static battery with/without Ch^+^ and SO_4_^2−^ electrolytes is illustrated schematically in Figure 3. Without Ch^+^ and SO_4_^2−^ electrolyte, the battery easily produces soluble Br_3_^−^ during charging, which directly leads to the pronounced shuttle effect (Figure 3a). In contrast, the introduction of Ch^+^ and SO_4_^2−^ electrolyte effectively mitigates the shuttle effect of Br_3_^−^, as shown in Figure 3b. The suppression of the shuttle effect originates from the synergistic effect of Ch^+^ and SO_4_^2−^. First, Ch^+^ with a high atomic charge of N^+^ site strongly attracts soluble Br_3_^−^, preventing their dissolution into aqueous electrolyte. Beyond the action of Ch^+^, the high negative charge density of SO_4_^2−^ creates strong electrostatic repulsion that further drives down the solubility of Br_3_^−^ in aqueous electrolyte.

**FIGURE 4:**
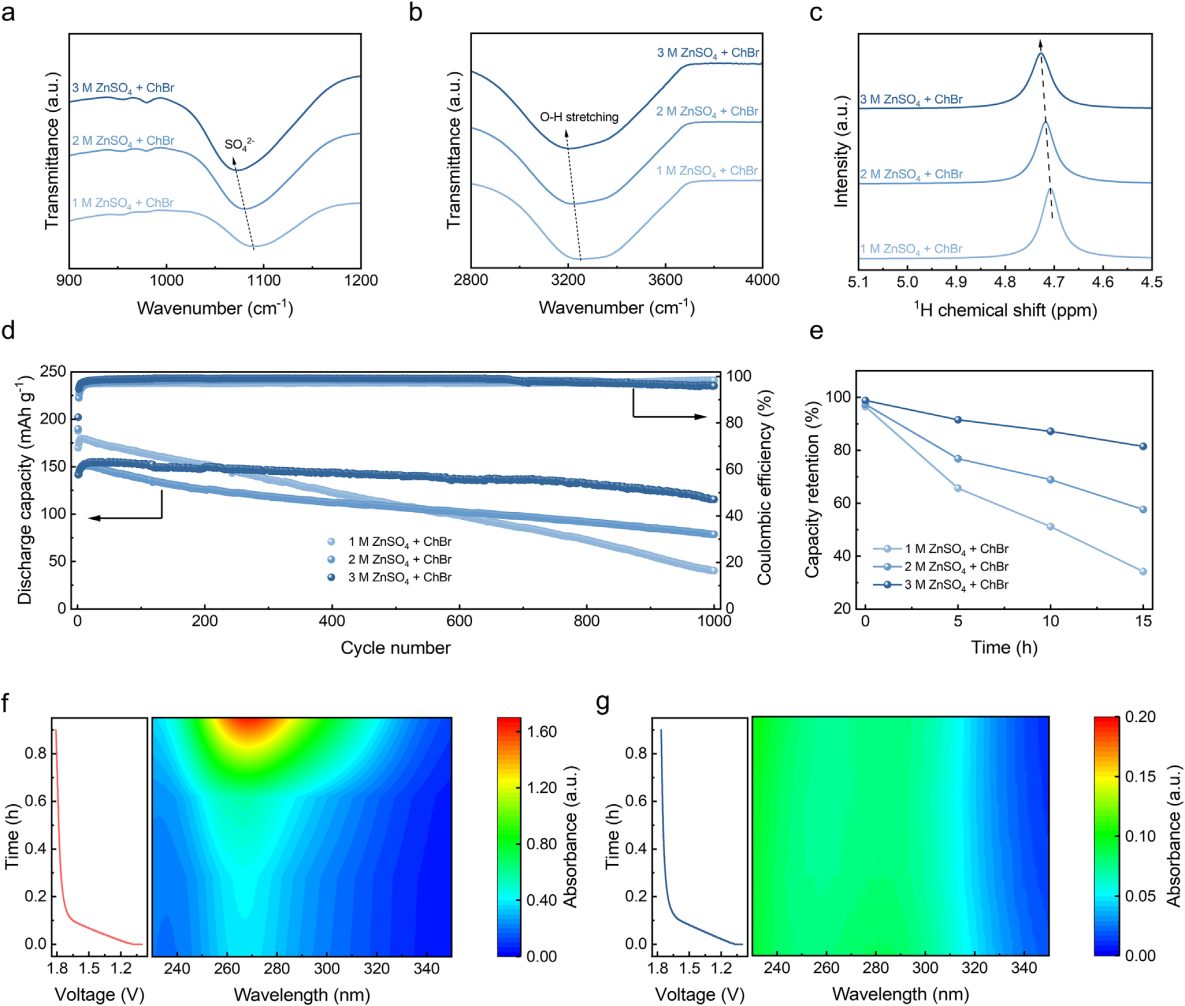
FT‐IR spectra in (a) low wavenumber and (b) high wavenumber. (c) ^1^H NMR spectra of various ZnSO_4_ concentration electrolytes. (d) Cycling performance of Zn–Br static battery with various ZnSO_4_ concentration electrolytes at a current density of 0.5 A g^−1^. (e) Capacity retention after self‐discharge 15 h of Zn–Br static battery with various ZnSO_4_ concentration electrolytes at a current density of 0.5 A g^−1^. In situ UV–vis spectra of Zn–Br static battery with (f) 1 m ZnSO_4_ + ChBr electrolyte and (g) 3 m ZnSO_4_ + ChBr electrolyte.

An optimal ZnSO_4_ concentration was identified to suppress H_2_O activity and enhance the reversibility of the cathode reaction [45]. First, Fourier transform infrared (FT‐IR) spectroscopy and proton nuclear magnetic resonance (^1^H NMR) spectroscopy were employed to investigate the influence of ZnSO_4_ concentration on the H─bond network. With increasing ZnSO_4_ concentration, the signal of SO_4_^2−^ (900–1200 cm^−1^) exhibits an obvious redshift (Figure 4a). Meanwhile, the O‐H stretching band (2800–4000 cm^−1^) shifts to a lower wavenumber (Figure 4b), implying the strong interaction between SO_4_^2−^ and H_2_O [46]. The finding is corroborated by ^1^H NMR spectra (Figure 4c), where the H_2_O peak shifts downfield from 4.71 to 4.73 ppm as ZnSO_4_ concentration increases. The original H─bond network among water molecules is disrupted, while the highly electronegative O atoms of SO_4_^2−^ attract H atoms of H_2_O, forming a new H─bond network and capturing free H_2_O (Figure S3). The salting‑out effect of a high concentration of SO_4_^2−^ significantly lowers water activity, which weakens the hydration shell of Br_3_^−^ and promotes its partitioning into the electrode. Meanwhile, the increased ionic strength lowers the activity of Br_3_^−^ through non‑ideal solution behavior, thereby reducing its effective thermodynamic driving force for dissolution and migration. Impressively, Zn–Br static battery with 3 m ZnSO_4_ electrolyte demonstrates superior reversibility, maintaining a high discharge capacity of over 1000 cycles with 81.5% of the initial capacity and an average CE of 98.4% (Figure 4d and Figure S4). In contrast, the batteries with low‐concentration ZnSO_4_ electrolytes exhibit lower discharge capacity and average CE after 1000 cycles. As shown in Figure 4e, the capacity retention of Zn–Br static battery with 3 m ZnSO_4_ electrolyte is significantly higher than that of 1 and 2 m ZnSO_4_ electrolytes after self‐discharge testing. Following a 5 h rest period, the battery with 1 m ZnSO_4_ electrolyte retains only 66% of its capacity, compared to 92% with the 3 m variant. Even after extending the rest period to 15 h, the capacity retention of the battery with 3 m ZnSO_4_ remains as high as 81%.

Employing in situ UV–vis spectroscopy, we characterized the Br_3_^−^ shuttle behavior in Zn–Br static batteries after charging (Figure 4f,g). A home‐made cell, which contains 1 m ZnSO_4_ electrolyte or 3 m ZnSO_4_ electrolyte, as well as activated carbon cathode and Zn anode on opposing sides of the irradiation path, facilitates the detection of any dissolution of Br_3_^−^ into the electrolyte (Figure S5). For 1 m ZnSO_4_ electrolyte, UV–vis spectra show a gradual increase in absorption peak intensity (Figure 4f), indicating that the Br_3_^−^ partially dissolves into the electrolyte. In contrast, UV–vis spectra for 3 m ZnSO_4_ electrolyte exhibits extremely low peak intensity (Figure 4g), indicating that the shuttle and dissolution of Br_3_^−^ is effectively suppressed. To probe the reaction mechanism, we further investigated the cathode evolution in 3 m ZnSO_4_ electrolyte. To verify the state of the generated Br_3_^−^, we mixed Br_3_^−^ with water or the optimized electrolyte. It is evident that the Br_3_^−^ readily dissolves in water, resulting in a noticeable yellow color (Figure S6). In sharp contrast, it was observed that Br_3_^−^ is confined to the top of the solution and keeps an oil phase in the presence of the optimized electrolyte, resulting in significant suppression of their diffusion. We therefore speculate that Zn–Br static battery with the 3 m ZnSO_4_ + ChBr electrolyte enables the rapid reaction kinetics in the cathode. Scanning electron microscopy (SEM) image and corresponding energy dispersive x‐ray spectroscopy (EDS) element distribution of the cathode reveals the formation of Br element during charging to 1.9 V (Figure S7), indicating effective immobilization of Br_3_^−^. Meanwhile, XPS spectra further demonstrate that Br_3_^−^ generate and disappear during charging and discharge process, respectively (Figure S8). Br_3_^−^ crossover not only affects the cathode side but also poses a serious threat to the Zn anode through corrosion and parasitic side reactions. Accordingly, we performed additional characterizations of the Zn anode after cycling. We examined the surface morphology of the Zn anode with different electrolytes after 50 cycles. As shown in Figure S9, the Zn electrode in the optimized electrolyte (3 m ZnSO_4_ + ChBr) maintains a relatively smooth and dense surface, with significantly less dendritic growth and fewer corrosion products compared to the control electrolytes. In addition, the diffraction peaks are well‐indexed to metallic Zn with no other detectable peaks in the optimized system (Figure S10). In contrast, the control sample (1 m ZnSO_4_ + KBr) shows an additional weak peak indicative of surface corrosion products. To quantitatively assess the corrosion resistance of the Zn anode in the optimized electrolyte, we performed Tafel measurements in different electrolytes. As shown in Figure S11, the corrosion current density decreases significantly in the optimized electrolyte compared to the control electrolytes. These results demonstrate that the optimized electrolyte provides enhanced protection against electrochemical corrosion. Therefore, 3 m ZnSO_4_ + ChBr electrolyte contributes to the reversibility of Zn–Br static battery, which is identified as the optimal condition for the following testing.

**FIGURE 5:**
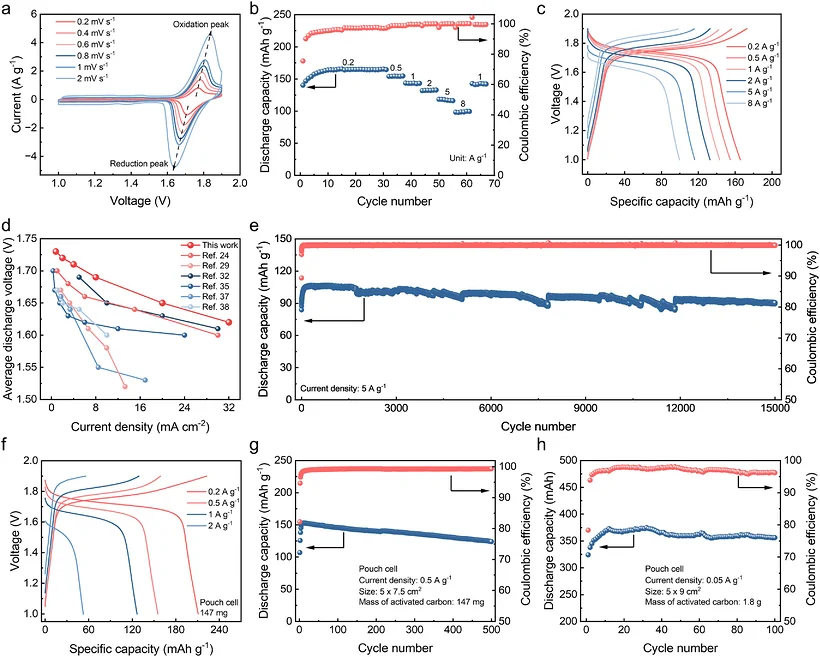
(a) CV curves of Zn–Br static battery at various scan rates ranging from 0.2 to 2 mV s^−1^. (b) Rate capability and (c) GCD curves of Zn–Br static battery at various current densities ranging from 0.2 to 8 A g^−1^. (d) Comparison of average discharge voltage in various current rates among the reported Zn–Br static batteries. (e) Cycling performance of Zn–Br static battery at a current density of 5 A g^−1^. (f) GCD curves of Zn–Br pouch cell at various current densities ranging from 0.2 to 2.0 A g^−1^. (g) Cycling performance of Zn–Br pouch cell with an electrode size of 5 cm × 7.5 cm at a current density of 0.5 A g^−1^. (h) Cycling performance of high‐loading Zn–Br pouch cell with an electrode size of 5 cm × 9 cm at a current density of 0.05 A g^−1^.

To assess the reaction kinetics of Zn–Br static battery, cyclic voltammetry (CV) measurements were conducted in various scan rates (Figure 5a). With the scan rate increasing from 0.2 to 2 mV s^−1^, CV curves keep the same shape and the oxidation peak shifts to high voltage while the reduction peak shifts to low voltage, indicating the reversible cathode reaction. By plotting log(*i*) vs. log(*v*), the linear fitted b value of reduction and oxidation peak is 0.64 and 0.75, respectively (Figure S12), indicating a typical battery system involving both diffusion‐controlled and capacitive‐controlled processes [47]. The rate capability and cycling performance of Zn–Br static battery with 3 m ZnSO_4_ + ChBr electrolyte were further evaluated. At current densities ranging from 0.2 to 8 A g^−1^ (0.2, 0.5, 1, 2, 5, and 8 A g^−1^), Zn–Br static battery delivers reversible capacities of 166, 155, 143, 133, 116, and 99 mAh g^−1^ (Figure 5b). When the current density returns to 1 A g^−1^, the battery capacity also recovers to 143 mAh g^−1^. This superior rate performance suggested facilitated reaction kinetics by Ch^+^ and SO_4_^2−^. As shown in Figure 5c, the corresponding GCD curves maintain well‐defined voltage plateaus across all tested current densities. The battery delivers high average discharge voltages of 1.73, 1.72, 1.71, 1.69, 1.65, and 1.62 V at the current densities of 0.2, 0.5, 1, 2, 5, and 8 A g^−1^, respectively. Meanwhile, the battery retains a high power density of 13 kW kg^−1^ at 8 A g^−1^. Compared with previous Zn–Br static batteries [24, 29, 32, 35, 37, 38], this study demonstrates significant advancement in current rate and average discharge voltage (Figure 5d and Table S1), highlighting the effectiveness of the electrolyte strategy based on the Br_3_^−^ attraction‐repulsion regulation. The cycling stability for Zn–Br static battery was first evaluated under 1 A g^−1^. The battery exhibits an initial capacity increase during several cycles. Unless otherwise stated, all values of capacity retention are normalized to the peak capacity. The battery achieves a reversible discharge capacity of 120 mAh g^−1^ and maintains a capacity retention of 85.8% after 2000 cycles (Figure S13). Meanwhile, the battery delivers a high‐capacity retention of 82.5% after 5000 cycles at 2 A g^−1^ (Figure S14). Even at a current density of 5 A g^−1^, the battery still achieves a capacity retention of 84.9% after 15 000 cycles and maintains an average CE of ∼100% (Figure 5e). The average capacity decay is approximately 0.0010% per cycle. The excellent stability of Zn–Br static battery with 3 m ZnSO_4_ + ChBr electrolyte is further confirmed by its stable GCD curves with negligible change in energy efficiency (89%) and voltage efficiency (89%) after 15 000 cycles (Figure S15).

To further verify the practical application of the designed electrolyte, Zn–Br pouch cells were assembled. The experimental setup includes a piece of AC cathode and a piece of Zn foil anode with an electrode size of 5 cm × 7.5 cm. The assembled pouch cells were used to test rate capability, cycling stability, and self‐discharge performance. As illustrated in Figure 5f, the pouch cell achieves the discharge capacities of 211, 155, 127, and 53 mAh g^−1^ at 0.2, 0.5, 1, and 2 A g^−1^, respectively. Upon returning to 1 A g^−1^, the discharge capacity of the battery only presents a slight change (Figure S16). As shown in Figure 5g and Figure S17, the Zn–Br pouch cell delivers impressive cycling stability with a capacity retention of 81.1% after 500 cycles at 0.5 A g^−1^. We further evaluated the self‐discharge behavior of the pouch cell that critically governs the calendar lifespan of Zn–Br static batteries. As shown in Figure S18, the capacity retention reaches to 71.4% after self‐discharge 15 h. Upon full charging, two pouch cells in series are capable of powering a light‐emitting diode (LED) screen, confirming its feasibility for practical applications (Figure S19). The high‐loading pouch cell with a practical areal capacity of 8.3 mAh cm^−2^ and an electrode size of 5 cm × 9 cm was designed to enhance the overall battery capacity. As shown in Figure 5h and Figure S20, the high‐loading pouch cell achieves a high discharge capacity of 356 mAh after 100 cycles at 0.05 A g^−1^. The Zn–Br static battery for high‐loading pouch cell delivers negligible change in energy efficiency (91%) and voltage efficiency (93%) after 100 cycles. These results validate the effectiveness of the proposed Br_3_^−^ attraction‐repulsion regulation in practical Zn–Br pouch cells, offering crucial guidance for the rational design of advanced electrolytes aimed at Zn–Br static battery applications.

## 3 Conclusion

In summary, we design a Br_3_^−^ attraction‐repulsion regulation relying on the synergistic effect of Ch^+^ and SO_4_^2−^, which effectively enhances the reversibility and reaction kinetics of Zn–Br static batteries. Theoretical calculations and experimental results indicate that Ch^+^ possess strong bonding effect in Br_3_^−^ and SO_4_^2−^ possess strong electrostatic repulsion on Br_3_^−^, thus preventing the Br_3_^−^ dissolving and shuttling into the electrolyte. Following the optimization of ZnSO_4_ concentration, the destruction of the H_2_O─H_2_O H─bond network can thereby boost battery reversibility. As a result, Zn–Br static battery delivers a high discharge capacity of 166 mAh g^−1^ and a high average discharge voltage of 1.73 V at 0.2 A g^−1^. Meanwhile, the Zn–Br static battery exhibits outstanding rate capability and cycling stability, including a discharge capacity of 99 mAh g^−1^ at a high rate of 8 A g^−1^ and 84.9% capacity retention over 15 000 cycles at 5 A g^−1^. More importantly, Zn–Br pouch cell with a high areal capacity of 8.3 mAh cm^−2^ can achieve a real capacity of 365 mAh after 100 cycles at 0.05 A g^−1^. This work, therefore, provides a promising blueprint for practical aqueous Zn–Br static batteries.

## Author Contributions

**Jiamin Tang**: writing – original draft, investigation, validation, data curation, formal analysis. **Ling He**: data curation, validation, investigation, formal analysis, Writing – original draft. **Lei Liu**: validation, visualization. **Huanwei Li**: validation, visualization. **Haichuan He**: validation, visualization. **Jie Wang**: validation, visualization. **Zhengxin Zhu**: methodology, writing – review and editing, investigation, funding acquisition, supervision, project administration.

## Conflicts of Interest

The authors declare no conflicts of interest.

## Supporting information




**Supporting File**: advs77018‐sup‐0001‐SuppMat.docx.

## Data Availability

The data that support the findings of this study are available from the corresponding author upon reasonable request.
