# Supplementary material for: Polybromides Attraction‐Repulsion Regulation Promotes High‐Performance Zinc–Bromine Static Battery
Source: Adv Sci (Weinh). 2026 Sep 6:e77018. Online ahead of print. doi: 10.1002/advs.77018 (PMC13547541; doi:10.1002/advs.77018)
Supplement: Supplementary file 1 [file ADVS-9999-e77018-s001.docx]

Supporting Information

Polybromides Attraction-Repulsion Regulation Promotes High-Performance Zinc-Bromine Static Battery

*Jiamin Tang, Ling He, Lei Liu, Huanwei Li, Haichuan He, Jie Wang, Zhengxin Zhu**

J. Tang, L. He, L. Liu, H. Li, H. He, J. Wang, Z. Zhu

School of Chemistry and Chemical Engineering, University of South China, Hengyang 421001, China
E-mail: [zhuzhengxin@usc.edu.cn](mailto:zhuzhengxin@usc.edu.cn)

Jiamin Tang and Ling He contributed equally to this work.

**1 Materials**

Zinc sulfate monohydrate (ZnSO_4_·H_2_O, 99%), zinc trifluoromethanesulfonate (Zn(OTf)_2_, ≥98%), zinc chloride (ZnCl_2_, ≥98%), zinc perchlorate hexahydrate (Zn(ClO_4_)_2_, 99.995%), bis (trifluromethanesulfonyl) imide zinc (Zn(TFSI)_2_, 98%), zinc acetate dihydra (Zn(Ac)_2_·2H_2_O, ≥99%), sodium bromide (NaBr, ≥99%), lithium bromide (LiBr, 99.5%), potassium bromide (KBr, ≥99%), choline bromide (ChBr, 98%), and ethanol were purchased from Shanghai Taitan Scientific Technology Co., Ltd. Activated carbon (AC, YP-50), acetylene black (AB), carboxymethyl cellulose sodium (CMC-Na), polytetrafluoroethylene (PTFE, 60%) solution, Zn foil (thickness: 100 μm), Ti mesh (80 mesh), and Ti foil (thickness: 20 μm), and glass fiber (GF/B, Whatman) were purchased from Jinghong New Energy Technology Co., Ltd. All aqueous electrolytes were prepared with deionized water.

**2 Preparation of electrodes**

For the low-loading AC cathode, the binder was previous prepared by the dissolution of CMC-Na in the deionized water. The cathode slurry was prepared by the mixture of AC, AB, and CMC-Na binder in a mass ratio of 8:1:1. Then, the prepared slurry was coated onto Ti foil with a mass loading of ~4 mg cm^-2^. Finally, the cathode dried at 55 ^o^C for at least 12 h. For the high-loading AC cathode, AC, AB, and PTFE were mixed in a mass ratio of 18:1:1, with ethanol/H_2_O employed to help better mixing. The mixture was then pressed onto Ti mesh with a mass loading of ~40 mg cm^-2^.

**3 Characterization techniques**

The H-bond structure of various electrolytes was analyzed by Fourier transform infrared (FT-IR) spectra (IRTracer-100) and proton nuclear magnetic resonance (^1^H NMR) spectra (Bruker 400 M). The energy dispersive X-ray spectroscopy (EDS) elements mapping of AC cathode was obtained with the combination of scanning electron microscope (SEM, Zeiss Sigma 300). The valent change of Br element was performed on X-ray photoelectron spectroscopy (XPS, Thermo Scientific ESCALAB Xi+). Ultraviolet-visible (UV-Vis) spectra of various electrolytes were obtained by using a TU-1901 spectrometer (Persee) within a wavelength range of 230-350 nm. For *ex situ* UV-Vis spectra, the tested electrolytes were collected in the beaker cells after 10 cycles. For *in situ* UV-Vis spectra, a home-made cell was prepared by placing AC cathode and Zn foil anode on the rough sides of a quartz cell, followed by the addition of 3 mL of 1 M ZnSO_4_ + 0.5 M ChBr or 3 M ZnSO_4_ + 0.5 M ChBr. During UV-Vis measurements, the galvanostatic charging and discharging of Zn-Br battery were conducted using an electrochemical workstation (CHI760F, Chenhua).

**4 Electrochemical measurements**

The tested Zn foil required a requires a pretreatment with ethanol and H_2_O in order to remove the residues. For the coin cell, the Zn-Br battery was assembled by glass fiber separator, Zn anode, and AC cathode in an electrode area of 1.13 cm^2^ with 100 μL of electrolyte. The Zn-Br pouch cell was assembled with Zn foil anode (5*7 cm^2^), AC cathode (5*7 cm^2^), and glass fiber separator (6*8 cm^2^) with 3.5 mL of electrolyte. For the high-loading pouch cell (40 mg cm^-2^), the battery was assembled with Zn foil anode (5*9 cm^2^), AC cathode (5*9 cm^2^), and glass fiber separator (6*10 cm^2^) with 10 mL of electrolyte (2 M ZnSO_4_ + 2 M ChBr). Based on the mass of activated carbon, the theoretical capacity is 197 mAh g^-1^, which is calculated by the Br amount in the electrolyte. The energy density (E) is calculated based on the equations: E = U_A_*C, Where the U_A_ is the average discharge voltage, C is the specific capacity (based on the mass of activated carbon). In the coin cell, the electrolyte-to-capacity ratio is 133 mL/Ah and the energy density is 287 Wh kg^-1^ (based on the mass of activated carbon). In the high-loading pouch cell, the electrolyte-to-capacity ratio is 28 mL/Ah and the energy density is 345 Wh kg^-1^ (based on the mass of activated carbon). Galvanostatic charge/discharge (GCD) tests were performed using a Neware battery testing system (CT-4008Tn-5V50mA-HWX) within a voltage range of 1-1.9 V. Linear sweep voltammetry (LSV) measurements were conducted in a three-electrode cell, using Ti foil as the working electrode, platinum foil as counter electrode, and Ag/AgCl as reference electrode at a scan rate of 5 mV s^-1^. Cyclic voltammetry (CV) and LSV tests were carried out on an electrochemical workstation (BioLogic).

**5 Calculation methods**

All calculations were conducted using the Gaussian 16, C01 software package. The SDD basis set was applied to Br atoms, while the 6-311+G(d,p) basis set was used for all other atoms, within the framework of the B3LYP functional incorporating Grimme’s DFT-D3 dispersion correction with Becke-Johnson damping (DFT-D3BJ). DFT calculations were performed on isolated molecular clusters in the gas phase to evaluate the intrinsic binding energies between Ch^+^ and Br_3_^-^, and between H_2_O and Br_3_^-^. The binding energy (E_bind_) between molecule A and molecule B was derived from the expression E_bind_ = E_complex_(AB) − (E_part_(A) + E_part(_B)). The diagram of Electrostatic Potential (ESP) was visualized with the help of the GaussView, Version 6.0 program 1 Material preparation.

**Figure S1.** GCD curves of Zn-Br static battery with various Zn-based electrolytes after self-discharge 5 h at a current density of 0.5 A g^-1^.

**Figure S2.** Digital photo of the tested Zn-based electrolytes after 10 cycles.

**Figure S3.** (a) Schematic diagram of H_2_O-H_2_O H-bond network. (b) Schematic diagram of H_2_O-SO_4_^2-^ H-bond network.

**Figure S4.** (a) GCD curves of 1^st^ cycle and (b) GCD curves of 1000^th^ cycle of Zn-Br static battery with various ZnSO_4_ concentration electrolytes at a current density of 0.5 A g^-1^.

**Figure S5.** *In situ* UV-Vis measurements were performed by assembling a home-made cell.

**Figure S6.** Photographs of the Br_3_^-^ immersed in pure water or 3 M ZnSO_4_ + ChBr solution.

**Figure** **S7.** (a) SEM image and (b,c) corresponding EDS element distribution of the cathode after charging with 3 M ZnSO_4_ + ChBr electrolyte.

**Figure S8.** XPS spectra of the cathode with 3 M ZnSO_4_ + ChBr electrolyte after one cycle.

**Figure S9.** SEM images of the Zn anode in (a) 1 M ZnSO_4_ + KBr, (b) 1 M Zn(ClO_4_)_2_ + ChBr, (c) 1 M ZnSO_4_ + ChBr, and (d) 3 M ZnSO_4_ + ChBr electrolytes after 50 cycles.

**Figure S10.** XRD spectra of the Zn anode in 1 M ZnSO_4_ + KBr, 1 M Zn(ClO_4_)_2_ + ChBr, 1 M ZnSO_4_ + ChBr, and 3 M ZnSO_4_ + ChBr electrolytes after 50 cycles.

**Figure S11.** Tafel plots of the Zn anode in 1 M ZnSO_4_ + KBr, 1 M Zn(ClO_4_)_2_ + ChBr, 1 M ZnSO_4_ + ChBr, and 3 M ZnSO_4_ + ChBr electrolytes after 50 cycles.

**Figure S12.** Linear fitted relating to the peak current versus square root of scan rate. Based on the equation of *i* = a*v*^b^, the reaction is a diffusion-controlled process when b value is 0.5, and if b value reaches to 1, the pseudo capacitance will dominate the charge and discharge process.

**Figure S13.** Cycling performance of Zn-Br static battery at a current density of 1 A g^-1^.

**Figure S14.** Cycling performance of Zn-Br static battery at a current density of 2 A g^-1^.

**Figure S15.** GCD curves of Zn-Br static battery for the 1^st^, 5000^th^, 10,000^th^, and 15,000^th^ cycles at a current density of 5 Ag^-1^.

**Figure S16.** Rate capability of Zn-Br static battery at various current densities ranging from 0.2 to 2 A g^-1^.

**Figure S17.** GCD curves of Zn-Br pouch cell with an electrode size of 5 cm × 7.5 cm for the 1^st^, 100^th^, 250^th^, and 500^th^ cycles at a current density of 0.5 Ag^-1^.

**Figure S18.** Self-discharge performance of Zn-Br pouch cell with an electrode size of 5 cm × 7.5 cm after resting 15 h at a current density of 0.5 A g^-1^.

**Figure S19.** Photograph of powered LED by two Zn-Br pouch cells with an electrode size of 5 cm × 7.5 cm.

**Figure S20.** GCD curves of Zn-Br high-loading pouch cell with an electrode size of 5 cm × 9 cm for the 1^st^, 20^th^, 50^th^, and 100^th^ cycles at a current density of 0.05 Ag^-1^.

**Table S1.** Comparison of average discharge voltage in various current rates with reported Zn-Br static batteries.

| Cathode | Electrolyte | Average discharge voltage  (V/mA cm^-2^) | Battery type | Reference |
| --- | --- | --- | --- | --- |
| KBr-Oc_4_NBr | 3 M ZnSO_4_ | 1.66/1.69, 1.64/3.38, 1.55/8.45, 1.53/16.9, 1.48/33.8 | Coin cell | Ref. [1] |
| Br_2_-exCOF | 3 M ZnSO_4_ | 1.67/1.66, 1.65/3.32, 1.61/6.64, 1.58/9.96, 1.52/13.28 | Swagelok cell | Ref. [2] |
| [bMlmB]Br_2_ | 1 M ZnSO_4_ + 1 M ZnBr_2_ | 1.69/5, 1.65/10, 1.63/20, 1.61/30 | Swagelok cell | Ref. [3] |
| TPABr_3_ | 1 M Zn(DFTFSI)_2_ | 1.7/0.3, 1.67/0.6, 1.65/1.5, 1.63/3, 1.62/6, 1.61/12, 1.6/24 | Coin cell | Ref. [4] |
| CMK-3 | 0.2 M TPABr + 0.5 M ZnBr_2_ | 1.7/1, 1.68/3, 1.66/6, 1.64/15, 1.6/30 | Swagelok cell | Ref. [5] |
| Ti_2_CT*_x_* MXene | 2.5 m ZnBr_2_ | 1.67/1, 1.65/2, 1.64/5, 1.6/10 | Box cell | Ref. [6] |
| Activated carbon | 3 M ZnSO_4_ + 0.5 M ChBr | 1.73/0.8,1.72/2, 1.71/4, 1.69/8, 1.65/20, 1.62/32, | Coin cell | This work |

**References**

1. X. Zhao, J. Hao, Q. Chen, et al., "Aqueous Zinc‐Bromine Battery with Highly Reversible Bromine Conversion Chemistry," *Angewandte Chemie International Edition* 64 (2025): e202502386, <https://doi.org/10.1002/anie.202502386>.

2. Y. Zhang, C. Wei, M.-X. Wu, et al., "A High-Performance COF-Based Aqueous Zinc-Bromine Battery," *Chemical Engineering Journal* 451 (2023): 138915, <https://doi.org/10.1016/j.cej.2022.138915>.

3. Y. Wu, C. Xu, C. Lei, et al., "Optimizing Bromine Complexation and Kinetics: a Bisimidazole Strategy for High‐Performance Zn‐Br Static Batteries," *Angewandte Chemie International Edition* 64 (2025): 202509293, <https://doi.org/10.1002/anie.202509293>.

4. S. Chen, S. Li, L. Ma, et al., "Asymmetric Anion Zinc Salt Derived Solid Electrolyte Interphase Enabled Long-Lifespan Aqueous Zinc Bromine Batteries," *Angewandte Chemie-International Edition* 63 (2024): e202319125, <https://doi.org/10.1002/anie.202319125>.

5. L. Gao, Z. Li, Y. Zou, et al., "A High-Performance Aqueous Zinc-Bromine Static Battery," *iScience* 23 (2020): 101348, <https://doi.org/10.1016/j.isci.2020.101348>.

6. J. Guo, G. Ma, G. Liu, C. Dai, and Z. Lin, "Ti_2_CT*_x_* MXene Cathode Host for Enhanced Zinc-Bromine Battery Performance," *Advanced Energy Materials* 14 (2024): 2304516, <https://doi.org/10.1002/aenm.202304516>.
